# Metagenomic Shotgun Analyses Reveal Complex Patterns of Intra- and Interspecific Variation in the Intestinal Microbiomes of Codfishes

**DOI:** 10.1128/AEM.02788-19

**Published:** 2020-03-02

**Authors:** Even Sannes Riiser, Thomas H. A. Haverkamp, Srinidhi Varadharajan, Ørnulf Borgan, Kjetill S. Jakobsen, Sissel Jentoft, Bastiaan Star

**Affiliations:** a Centre for Ecological and Evolutionary Synthesis, Department of Biosciences, University of Oslo, Oslo, Norway; b Department of Mathematics, University of Oslo, Oslo, Norway; Centers for Disease Control and Prevention

**Keywords:** Atlantic cod, *Gadus morhua*, codfishes, intestinal microbiome, metagenomics

## Abstract

The relative importance of host-specific selection or environmental factors in determining the composition of the intestinal microbiome in wild vertebrates remains poorly understood. Here, we used metagenomic shotgun sequencing of individual specimens to compare the levels of intra- and interspecific variation of intestinal microbiome communities in two ecotypes (NEAC and NCC) of Atlantic cod (Gadus morhua) that have distinct behavior and habitats and three Gadidae species that occupy a range of ecological niches. Interestingly, we found significantly diverged microbiomes among the two Atlantic cod ecotypes. Interspecific patterns of variation are more variable, with significantly diverged communities for most species’ comparisons, apart from the comparison between coastal cod (NCC) and Norway pout (Trisopterus esmarkii), whose community compositions are not significantly diverged. The absence of consistent species-specific microbiomes suggests that external environmental factors, such as temperature, diet, or a combination thereof, comprise major drivers of the intestinal community composition of codfishes.

**IMPORTANCE** The composition of the intestinal microbial community associated with teleost fish is influenced by a diversity of factors, ranging from internal factors (such as host-specific selection) to external factors (such as niche occupation). These factors are often difficult to separate, as differences in niche occupation (e.g., diet, temperature, or salinity) may correlate with distinct evolutionary trajectories. Here, we investigate four gadoid species with contrasting levels of evolutionary separation and niche occupation. Using metagenomic shotgun sequencing, we observed distinct microbiomes among two Atlantic cod (*Gadus morhua*) ecotypes (NEAC and NCC) with distinct behavior and habitats. In contrast, interspecific patterns of variation were more variable. For instance, we did not observe interspecific differentiation between the microbiomes of coastal cod (NCC) and Norway pout (*Trisopterus esmarkii*), whose lineages underwent evolutionary separation over 20 million years ago. The observed pattern of microbiome variation in these gadoid species is therefore most parsimoniously explained by differences in niche occupation.

## Abstract (precis)

The composition of the intestinal microbial community associated with teleost fish is influenced by a diversity of factors, ranging from internal factors (such as host-specific selection) to external factors (such as niche occupation). These factors are often difficult to separate, as differences in niche occupation (e.g., diet, temperature, or salinity) may correlate with distinct evolutionary trajectories. Here, we investigate four gadoid species with contrasting levels of evolutionary separation and niche occupation. Using metagenomic shotgun sequencing, we observed distinct microbiomes among two Atlantic cod (*Gadus morhua*) ecotypes (NEAC and NCC) with distinct behavior and habitats. In contrast, interspecific patterns of variation were more variable. For instance, we did not observe interspecific differentiation between the microbiomes of coastal cod (NCC) and Norway pout (*Trisopterus esmarkii*), whose lineages underwent evolutionary separation over 20 million years ago. The observed pattern of microbiome variation in these gadoid species is therefore most parsimoniously explained by differences in niche occupation.

## INTRODUCTION

Significant research effort has focused on the importance of external, environmental factors (e.g., habitat, geography, microbial biodiversity, diet, water temperature, or salinity) and internal, host-related factors (e.g., genetics, physiology or immunity) in driving the composition of the intestinal microbiome in fish (1, 2). That external factors play an important role is well established. For instance, bacterial diversity in the surrounding water influences the intestinal microbiome in fish larvae and fry (3, 4); water temperature is the main driver for the gut microbiome composition in farmed Tasmanian Atlantic salmon (Salmo salar) (5); and diet influences the intestinal composition in both experimental (6–9) as well as wild fish populations (10–13). Yet internal factors also influence the composition of these bacterial communities. For instance, observations of a shared (core) microbiome between wild and laboratory-raised zebrafish suggest that distinct selective pressures determine the composition of the microbial communities (14). Moreover, an association between host phylogeny and intestinal microbiome composition has been observed for a range of fishes, marine animals, and terrestrial mammals (15–19).

The adaptive immune system appears especially important for host selection. Individual variation of the major histocompatibility complex (MHC) II correlates with the gut microbiome composition in stickleback (20); mucosal IgT depletion causes dysbiosis in rainbow trout (Oncorhynchus mykiss) (21); and lack of a functional adaptive immune system reduces the strength of host selection in knockout zebrafish models (22). Among bony fish, gadoid fishes have an unusual adaptive immune system in which there is loss of MHC II, CD4, and invariant chain (Ii) and in which there is a range of innate (TLR) and MHC I immune-gene expansions (23, 24). Moreover, Atlantic cod has high levels of IgM (25) and a minimal antibody response after pathogen exposure (25–27). Gadoids therefore provide an interesting ecological system to study host-microbiome interactions (28).

Studies that specifically integrate internal and external influences support a role for both factors driving the microbial community composition (13, 29). Such studies, however, remain restricted in both the level of taxonomy of fishes (30) as well as taxonomical resolution of the microbial analyses (16S rRNA) (13, 29, 31–33). Importantly, it often remains difficult to separate the correlated effects of distinct behavior (e.g., diet) and niche occupation with interspecific selection. Also, no comparative studies have used metagenomic shotgun sequencing to investigate fish populations with profound differences in behavior within a single species. It therefore remains unclear whether the microbial composition for a range of wild fish species is characterized by intra- or interspecific divergence.

Here, we study intra- and interspecific divergence of intestinal microbial communities within the widespread family of Gadidae using a metagenomic shotgun data set. We compare the microbiomes from Norway pout (*Trisopterus esmarkii*), poor cod (Trisopterus minutus), northern silvery pout (Gadiculus thori), and two ecotypes of Atlantic cod (*Gadus morhua*). These four species have overlapping geographical distributions, are dietary generalists in typically feeding over sandy and muddy bottoms on pelagic or benthic crustaceans, polychaetas, and (small) fish (34, 35), and evolutionarily diverged approximately 20 million years ago (24). Norway pout is benthopelagic, distributed from the English Channel, around Iceland, and up to the Southwest Barents Sea. It is mostly found at depths between 100 and 200 m. Poor cod is also benthopelagic, distributed from the Trondheim Fjord in Norway to the Mediterranean Sea, and mostly found between 15 and 200 m. Northern silvery pout (*Gadiculus thori*) is meso- to bathypelagic (36), distributed in the North Atlantic Ocean, along the coast of Norway, and around Iceland and Greenland. It forms large schools that are usually found between 200 and 400 m (34, 36, 37). Finally, Atlantic cod has a trans-Atlantic distribution, from the Bay of Biscay to the Barents Sea, the Baltic Sea, around Iceland and Greenland, in the Hudson Bay, and along the North American coast (34, 38–42). Atlantic cod comprises various subpopulations and “ecotypes” with distinct adaptations, migratory behavior, and feeding behavior. For instance, northeast Arctic cod (NEAC) performs typical spawning migrations from the Barents Sea to the Norwegian coast, whereas the Norwegian coastal cod (NCC) remains more stationary (34, 43). These ecotypes have increased genomic divergence in several large chromosomal inversions (43–47), suggestive of local adaptation. The environments that these two ecotypes encounter are different, and they feed on distinct types of food. NEAC consumes mostly capelin and herring while NCC feeds on a wide range of crustaceans, fish, and seaweed (34, 39, 48). During spawning, these ecotypes spatially co-occur, and long-term gene flow between ecotypes is supported by low overall estimates of divergence in most genomic regions, apart from the chromosomal rearrangements (43).

We hypothesize that if interspecific selection (indicative of host selection) is the main driver for the intestinal communities in the Gadidae, most differences will be found between the different species, and not between the different ecotypes within Atlantic cod. In contrast, if environmental factors are the main drivers for the intestinal communities, we expect significant compositional differences between the ecotypes of Atlantic cod, as well as various levels of differentiation between the species. We used taxonomic profiling of metagenomic shotgun reads to classify these microbiomes (obtained from various locations around the Norwegian coast [Table 1]) at order- and species-level resolution and analyzed within-species differentiation of the most abundant members by genome-wide single nucleotide variation. Finally, differences in gut bacterial community composition among the species and ecotypes were assessed using multivariate statistics.

**TABLE 1:** Species collected and sample locations

Species	Latin name	Ecotype	Sampling location	*n*	Abbreviation
Atlantic cod	*Gadus morhua*	Northeast Arctic cod	Lofoten	10	NEAC
Atlantic cod	*Gadus morhua*	Norwegian coastal cod	Lofoten	10	NCC
Atlantic cod	*Gadus morhua*	Norwegian coastal cod	Oslo fjord	2	NCC_Oslo
Poor cod	*Trisopterus minutus*		Oslo fjord	5	PC
Norway pout	*Trisopterus esmarkiii*		Oslo fjord	4	NP
Northern silvery pout	*Gadiculus thori*		Oslo fjord	3	NSP

## RESULTS

### Taxonomical composition of the intestinal microbiomes.

**FIG 1:**
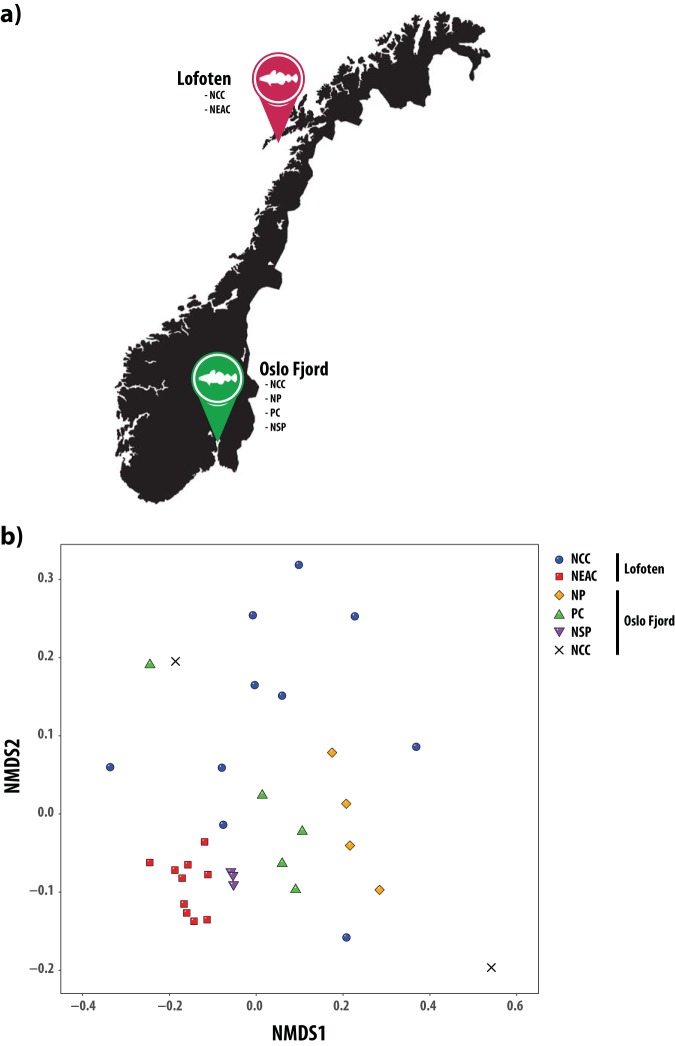
The intestinal microbiomes obtained from a range of gadoid species and ecotypes. (A) Map of sampling locations in Norway, Europe. Northeast Arctic cod (NEAC), and Norwegian coastal cod (NCC) were obtained from Lofoten. NCC (two individuals), poor cod (PC), Norway pout (NP), and northern silvery pout (NSP) were obtained from the Oslo Fjord. (Map data copyright 2020 Google.) (B) Nonmetric multidimensional scaling (NMDS) plot of nonnormalized, order-level sequence counts from the intestinal microbiomes of all samples. Each point represents an individual sample, and the species or ecotypes are indicated by different shapes and colors. The stress value of the NMDS plot is 0.14.

**FIG 2:**
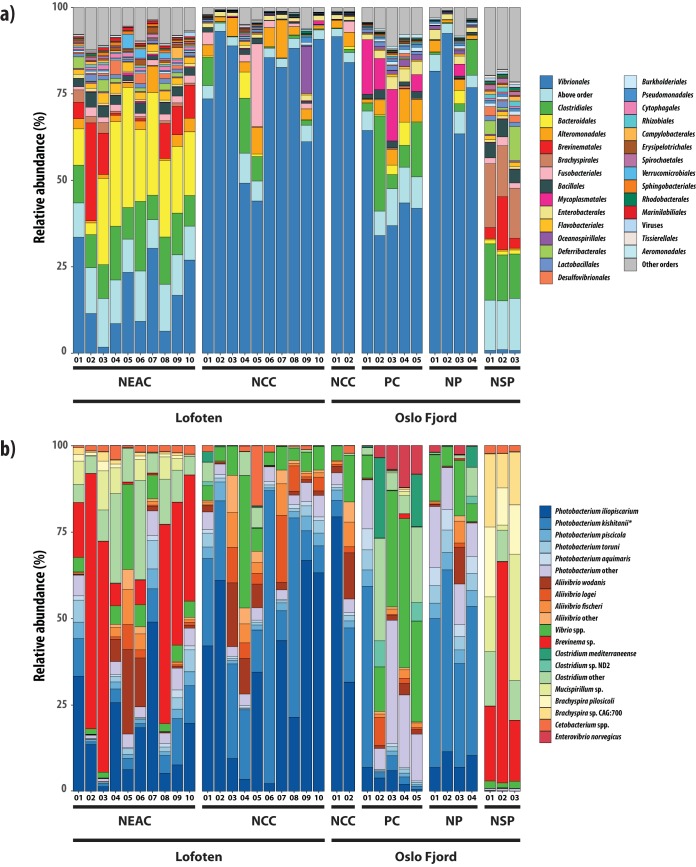
Taxonomic composition of the fish intestinal microbiomes. (a) Relative abundance of metagenomic shotgun sequences classified at the order level (93%). Colors represent the 28 orders with highest relative abundance, sequences assigned to other orders or viruses, and sequences classified above order level. Numbers along the *x* axis indicate the individual samples of the different species/ecotypes. (b) Relative abundance of metagenomic shotgun sequences classified at the species level (66%). The plot includes the most highly abundant species and other members of their parent bacterial genera (“other” categories) in the different fish species/ecotypes. Numbers along the *x* axis indicate the individual samples of the different species/ecotypes. The asterisk denotes the *P. kishitanii* strain that was reclassified from *P. phosphoreum*.

**FIG 3:**
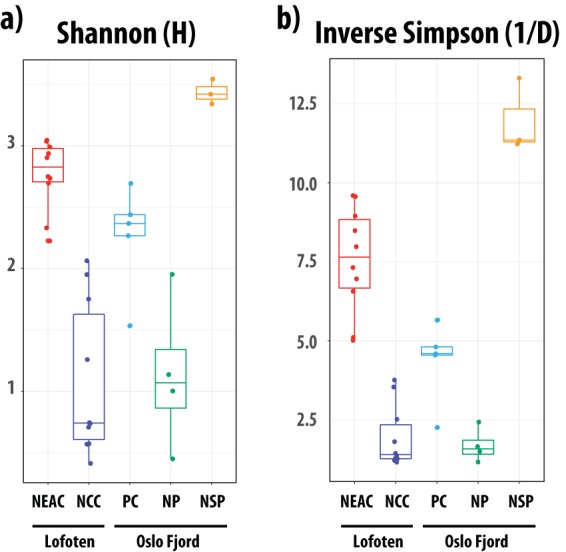
Within-sample microbial diversity in the gadoid species and ecotypes. Boxplots of Shannon (a) and inverse Simpson (b) diversity in the fish species/ecotypes. Each individual is represented by a point, and the individuals are grouped and colored by species and ecotype. The middle band represents the median, while the upper and lower bands show the 75^th^ and 25^th^ percentiles. The boxplots include the minimum and maximum alpha diversity values.

We analyzed a data set of 422 million paired-end reads, with a median sample size of 11.9 million reads (8.0 to 19.6 million reads per sample) (Table 2, Table S7 in the supplemental material). Following filtering, order-level classification could be obtained for 93% of all sequences (Table 2). Based on nonnormalized order-level sequence counts, we observed clear patterns of separation between species and ecotypes in a multivariate nonmetric multidimensional scaling (NMDS) plot (Fig. 1b), with NEAC and northern silvery pout forming distinct clusters, whereas the NCC populations encompass the Norway pout and poor cod populations. *Vibrionales* was the most abundant order detected in the intestinal microbiomes of NCC specimens at both coastal locations (mean relative abundance [MRA]: 76%) as well as Norway pout (MRA: 79%) and poor cod (MRA: 44%) (Table 3, Fig. 2a), with the remainder of each gut community consisting of a mix of orders with low relative abundance. The intestinal microbiomes of the NEAC and northern silvery pout specimens had a significantly more diverse community composition (Fig. 3, Fig. 2a). NEAC was dominated by *Bacteroidales* (MRA: 21%), *Vibrionales* (MRA: 17%), *Clostridiales* (MRA: 12%), and *Brevinematales* (MRA: 7%), while northern silvery pout had a high relative abundance of orders *Brachyspirales* (MRA: 16%) and *Clostridiales* (MRA: 14%). Distinct from the gut communities of the other fish populations, northern silvery pout had a low abundance of *Vibrionales*. Finally, the amount of sequences in the “Others” category, as well as sequences classified above order level (mean of all samples was 7.8%), varied slightly between the fish species (Table S8). A species-level classification was obtained for 66% of all sequences. Overall, species of the genus *Photobacterium* comprised on average 40.6% of the classified sequences, ranging from 0.2% in northern silvery pout to 74.3% in Norway pout (Fig. 2b). In particular, P. kishitanii and P. iliopiscarium represented on average 43% and 36% of all *Photobacterium* species, respectively, although the ratio differed in the different fish species (e.g., 49% versus 41% in NCC; 16% versus 56% in NEAC; and 55% versus 12% in Norway pout).

**TABLE 2:** Overview of individual metagenomic sequence data from gadoid intestines^*a*^

Sample	Raw reads	After quality trimming/filtering (%)	Host DNA (%)	Bacterial DNA (%)	Final reads
NCC_01	10,883,740	85.9	87.3	12.7	1,187,649
NCC_02	11,140,950	87.9	62.2	37.8	3,699,538
NCC_03	9,891,322	90.2	41.2	58.8	5,249,515
NCC_04	10,587,865	86.9	85.2	14.8	1,364,663
NCC_05	8,423,091	89.1	57.7	42.3	3,171,737
NCC_06	10,879,319	89.6	30.5	69.5	6,772,948
NCC_07	10,082,237	91.8	31.3	68.7	6,361,506
NCC_08	9,114,703	87.3	80.5	19.5	1,549,210
NCC_09	11,105,189	89.1	62.2	37.8	3,733,846
NCC_10	11,140,743	84.7	86.0	14.0	1,320,875
NEAC_01	13,120,072	89.7	53.6	46.4	5,463,098
NEAC_02	12,119,926	89.6	56.8	43.2	4,687,565
NEAC_03	11,981,093	89.2	54.4	45.6	4,869,722
NEAC_04	12,618,529	91.1	33.5	66.5	7,646,256
NEAC_05	12,154,047	87.6	74.2	25.8	2,747,042
NEAC_06	13,883,762	88.4	59.5	40.5	4,971,507
NEAC_07	12,149,049	89.1	56.9	43.1	4,666,533
NEAC_08	11,861,852	88.7	64.0	36.0	3,787,155
NEAC_09	11,131,413	85.7	75.1	24.9	2,378,591
NEAC_10	15,483,018	83.7	82.9	17.1	2,221,214
OO_cod_01	8,047,125	83.7	85.9	14.1	949,039
IO_cod_01	9,716,392	90.2	43.7	56.3	4,937,260
NP_01	15,621,734	84.7	51.6	48.4	6,400,120
NP_02	16,548,297	90.2	18.1	81.9	12,224,903
NP_03	16,608,312	78.3	72.6	27.4	3,568,206
NP_04	13,459,929	83.0	61.5	38.5	4,300,178
PC_01	10,743,586	87.7	30.5	69.5	6,550,868
PC_02	18,982,339	81.0	29.6	70.4	10,833,201
PC_03	9,420,298	84.1	54.9	45.1	3,568,861
PC_04	9,623,591	87.7	30.0	70.0	5,908,283
PC_05	19,630,680	77.7	55.0	45.0	6,861,474
NSP_01	14,283,994	73.2	67.5	32.5	3,396,962
NSP_02	14,527,770	76.3	63.8	36.2	4,008,926
NSP_03	15,261,446	80.9	66.6	33.4	4,123,131
**Total:**	**422,227,413**				**155,481,582**
**Mean:**	**12,418,453**	**86.0**	**57.8**	**42.2**	

a PhiX- and human-derived DNA sequences represented a negligible proportion and were excluded from the table. On average, 42.2% of the quality filtered reads per sample were used for microbiome analysis. (For further details, see Table S7.)

**TABLE 3:** Mean relative abundance (%) of the ten most abundant bacterial orders in the intestinal microbiomes of gadoid species and ecotypes^*a*^

NEAC^*b*^	NCC^*b*^	PC	NP	NSP
*Bacteroidales* (21.39)	*Vibrionales* (75.69)	*Vibrionales* (44.13)	*Vibrionales* (78.57)	*Brachyspirales* (15.88)
*Vibrionales* (16.83)	*Alteromonadales* (4.34)	*Clostridiales* (11.16)	*Clostridiales* (3.36)	*Clostridiales* (14.14)
*Clostridiales* (11.66)	*Clostridiales* (3.47)	*Mycoplasmatales* (8.92)	*Alteromonadales* (2.11)	*Brevinematales* (7.11)
*Brevinematales* (7.43)	*Fusobacteriales* (3.46)	*Alteromonadales* (5.15)	*Enterobacterales* (2.04)	*Deferribacterales* (4.81)
*Bacillales* (2.64)	*Oceanospirillales* (1.56)	*Enterobacterales* (2.97)	*Bacteroidales* (1.02)	*Bacillales* (4.44)
*Alteromonadales* (2.61)	*Enterobacterales* (1.19)	*Bacteroidales* (2.09)	*Mycoplasmatales* (0.87)	*Fusobacteriales* (1.94)
*Flavobacteriales* (2.17)	*Bacteroidales* (0.92)	*Bacillales* (1.81)	*Oceanospirillales* (0.64)	*Desulfovibrionales* (1.70)
*Fusobacteriales* (1.62)	*Bacillales* (0.60)	*Oceanospirillales* (1.18)	*Burkholderiales* (0.47)	*Lactobacillales* (1.57)
*Brachyspirales* (1.23)	*Pseudomonadales* (0.33)	*Lactobacillales* (0.95)	*Bacillales* (0.43)	*Rhizobiales* (1.40)
*Deferribacterales* (1.23)	*Flavobacteriales* (0.27)	*Burkholderiales* (0.84)	*Pseudomonadales* (0.43)	*Spirochaetales* (1.28)

a NEAC, Northeast Arctic cod; NCC, Norwegian coastal cod; PC, poor cod; NP, Norway pout; NSP, northern silvery pout.

b These two ecotypes belong to the same species, *Gadus morhua*.

The NCC Lofoten intestinal microbiome was dominated by *P. iliopiscarium* (MRA: 21%) and *P. kishitanii* (MRA: 20%), followed by different species of *Aliivibrio* (A. wodanis, A. logei, and A. fischeri) (MRA: 13%) (Fig. 2b). Similarly, the bacterial gut community of Norway pout was also dominated by *Photobacterium* species, in particular *P. kishitanii* (MRA: 17%). The intestinal microbiome of poor cod was dominated by *Photobacterium* species (MRA: 18%), followed by different *Vibrio* spp. (MRA: 8%). The gut bacterial community of NEAC was more diverse, with high relative abundance of a *Brevinema* sp. (MRA: 31%) and different species in the genera *Photobacterium* (MRA: 34%), *Clostridium* (MRA: 12%), and *Aliivibrio* (MRA: 9%). The high abundance of *Bacteroidales* observed at the order level (Fig. 2a) was not reflected at the species level, as this order represented a high number of *Bacteroidales* species with low abundance. Consequently, no *Bacteroidales* species were among the 15 most abundant species in the NEAC intestinal microbiome (Fig. 2b). The NEAC samples also contained a *Mucispirillum* sp. (MRA: 4%) and two *Brachyspira* spp. (MRA: 2%). In northern silvery pout, the gut microbiome was quite evenly distributed between the *Brevinema* sp., the *Mucispirillum* sp., Brachyspira pilosicoli, *Brachyspira* sp. CAG:700, and a group of different *Clostridium* species in two of three samples. The third sample contained the same species but had an even higher relative abundance of the *Brevinema* sp. (64%) (Fig. 2b).

### Variation in bacterial community composition among species and ecotypes.

Significant differences in within-sample diversity (alpha diversity) at the order level were observed among all species and within-species ecotypes, except between NCC and Norway pout (Table 4, Table S5). None of the other covariates had a significant effect on alpha diversity. Similar to the results from the within-sample diversity, significant differences in community structure (beta diversity) were observed among the gadoid species at order, genus, and species levels (Table 5, Table S6). At the order level, the NEAC intestinal community had a different structure than what was observed in all the other gadoids (at a significance level of 0.05). The NCC intestinal microbiome was also different from that of both poor cod and northern silvery pout. In agreement with results of within-sample (alpha) diversity, no differences in community structure were observed between the microbiomes of NCC and Norway pout. Finally, no differences were observed between the gut microbiome of poor cod versus Norway pout, poor cod versus northern silvery pout, or Norway pout versus northern silvery pout (*P* = 0.074 for all). Beta diversity analysis also demonstrated that community differences at the genus and species levels were similar to those observed at the order level (Table S6).

**TABLE 4:** Effects of covariates on the intestinal microbial diversity (alpha diversity) of gadoid species and ecotypes^*a*^*^,^*^*b*^

Species or ecotype	Shannon		Simpson		Inv. Simpson	
	Estimate	*P* value	Estimate	*P* value	Estimate	*P* value
Intercept	1.08	**0.0000**	0.38	**0.0000**	1.93	**0.0001**
NEAC	1.69	**0.0000**	0.48	**0.0000**	5.62	**0.0000**
NP	0.06	0.8367	-0.01	0.8784	-0.25	0.7476
PC	1.18	**0.0001**	0.37	**0.0002**	2.44	**0.0017**
NSP	2.36	**0.0000**	0.53	**0.0000**	10.03	**0.0000**

a Results from the optimal linear regression models used in testing for significant effects of covariates on within-sample (alpha) diversity based on nonnormalized, order-level sequence counts. Population (species/ecotype) is the only covariate with a significant effect, and estimates are given relative to NCC. Significant effects (*P* < 0.05) are indicated in bold.

b NEAC, Northeast Arctic cod; PC, poor cod; NP, Norway pout; NSP, northern silvery pout.

**TABLE 5:** PERMANOVA analysis of intestinal microbial diversity from gadoid species and ecotypes^*a*^

Populations^*b*^	R^2^	*P* value	Adjusted *P* value
NEAC vs. NCC	0.71	**0.0001**	**0.0005**
NEAC vs. PC	0.53	**0.0002**	**0.0018**
NEAC vs. NP	0.70	**0.0010**	**0.0076**
NEAC vs. NSP	0.50	**0.0041**	**0.0207**
NCC vs. PC	0.42	**0.0026**	**0.0182**
NCC vs. NP	0.04	0.7138	0.7138
NCC vs. NSP	0.80	**0.0035**	**0.0207**
PC vs. NP	0.53	**0.0233**	0.0740
PC vs. NSP	0.80	**0.0185**	0.0740
NP vs. NSP	0.94	**0.0286**	0.0740

a R^2^ values, *P* values, and adjusted *P* values for pairwise comparisons of community composition (beta diversity) between the different species or ecotypes using PERMANOVA. The tests are based on Bray-Curtis dissimilarity calculated from order-level, normalized sequence counts. *P* values are adjusted for multiple testing by the Holm method. Significant differences (*P* < 0.05) are indicated in bold. (Genus- and species-level results can be found in Table S6.)

b NEAC, Northeast Arctic cod; NCC, Norwegian coastal cod; PC, poor cod; NP, Norway pout; NSP, northern silvery pout.

Differences in the intestinal community composition between these gadoids are predominantly explained by changes in the relative abundance of a limited number of orders. For example, different proportions of *Vibrionales* contribute 29% to the (Bray-Curtis) dissimilarity between the NCC and NEAC (*P* = 0.001), followed by differences in the relative abundance of *Bacteroidales*, explaining 10% of the dissimilarity (*P* = 0.001) (Table S9). Together, 80% of the observed dissimilarity between NCC and NEAC is explained by differences in their relative abundance of the top six orders. Similarly, 60% of the dissimilarity between NCC and northern silvery pout is driven by *Vibrionales*, *Brachyspirales*, and *Clostridiales*.

### Bacterial within-species variation of SNV heterogeneity.

**FIG 4:**
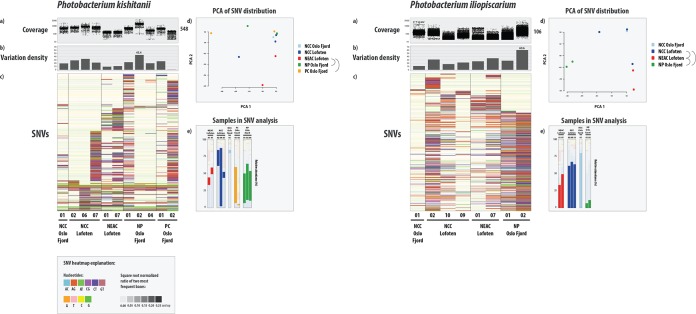
SNV variation analysis of the two most abundant bacterial genomes in the microbiomes of gadoid species. For each genome, the figure displays read coverage per single nucleotide variant (SNV) position in each sample from the different species/ecotypes (a) (mean coverage on right side of plot) and variation density (b) (number of variable positions per 1,000 bp reported in each individual sample, independent of coverage in the other samples) per sample (maximum value indicated). The *y* axes of the coverage and variation density plots are scaled across the genomes. (c) Heat map of a randomly chosen subset of 400 SNVs. In the heat map, each row represents a unique variable nucleotide position, where the color of each tile represents the two most frequent competing nucleotides in that position. The shade of each tile represents the square root-normalized ratio of the most frequent two bases at that position (i.e., the more variation in a nucleotide position, the darker the tile is). See legend at the bottom of the figure. (d) Principal-component analysis (PCA) plot of the SNV distribution (within-species variation) among the different samples. Each sample is represented by a dot, and colored according to species or ecotype membership. Half-circles to the right of the legend indicate species or ecotypes with significantly different within-species variation (i.e., different strains). (e) Relative abundance of the different samples used in variation analysis. The bars are colored according to the SNV plot in panel d.

We investigated bacterial within-species variation of *P. iliopiscarium* and *P. kishitanii*—with sufficient read coverage across all samples—among the different gadoids by mapping sequencing reads to their respective reference genomes (GCF_000949935.1 and GCF_000613045.2). In the samples used for single nucleotide variant (SNV) analysis, the mean percentage of the reference genomes with minimum 20-fold coverage (coverage breadth) after mapping were 63% for *P. iliopiscarium* and 19% for *P. kishitanii*. Hence, the variation analysis of the two species was based on different proportions of the reference genomes. The two reference genomes varied widely in the number of SNVs observed in all samples, from 84,866 in *P. iliopiscarium* to 1,229 in *P. kishitanii* (Fig. 4a). The density of variable sites within each individual sample showed various levels of heterogeneity in the bacterial populations (Fig. 4b). This heterogeneity was particularly clear in *P. kishitanii*, with site density varying from 0.5 to 45.4 variant positions per kbp per individual specimen. Further, the heat map showed gadoid-specific SNV patterns (Fig. 4c), in particular for *P. iliopiscarium*, where Norway pout contained a distinct pattern compared to the other gadoids, indicating the presence of specific *P. iliopiscarium* strain(s). Statistical analyses of SNV variation revealed that NEAC had a significantly different SNV pattern from Norway pout (Chi-square, *P* = 0.017) and poor cod (*P* = 0.028) for *P. kishitanii*, and from NCC (*P* = 0.033) and Norway pout (*P* = 0.000) for *P. iliopiscarium* (Fig. 4d, Table S10). NCC had a significantly different SNV pattern from Norway pout (*P* = 0.003) for *P. iliopiscarium*. (Fig. 4d, Table S10). The relative abundance of *P. kishitanii* and *P. iliopiscarium* varied greatly among the fish specimens used in the variation analysis (Fig. 4e).

## DISCUSSION

Using metagenomic shotgun sequencing, we show the composition of the intestinal microbiomes of two Atlantic cod ecotypes (NEAC, NCC) to be at least as divergent as those found between the different codfish species investigated here. Our findings have several implications for our understanding of the composition of the intestinal microbiome in wild fish populations.

Although species-specific selection has been proposed as a factor driving the composition of the intestinal community in fish in a variety of settings (13, 14, 16–19, 29, 33), our results show that this may not be the most important driver among gadoid species in wild populations. First, we observed highly significant differences in the intestinal microbiomes at order, species, and within-species bacterial levels between the NEAC and NCC ecotypes. Despite showing different migratory behaviors, these ecotypes co-occur during seasonal spawning in northern Norway (Lofoten), from where most of the samples were collected (43–45). Second, we observed no significant bacterial order- or species-level differences in the intestinal microbiome between different gadoid species, Atlantic cod (ecotype NCC), and Norway pout, which were sampled from different geographical locations (Lofoten and Oslo Fjord). We did not observe differentiation between the NCC sampled from Lofoten and the Oslo Fjord (although statistical certainly was low), which reflects an earlier observed lack of geographical structure for this ecotype (30). The similarity of the microbial compositions of the NCC and Norway pout is striking, as these are distinctly different genetic lineages with an evolutionary separation of at least 20 million years (24). These results suggest that NCC and Norway pout occupy an environmental niche that allows bacterial members with a broad geographical distribution to colonize their intestinal communities. Overall, the observation of a significant differentiation between microbiomes from ecotypes of the same species and a lack of differentiation between microbiomes from two distinct species suggests that the intestinal microbiome in these gadoid species and ecotypes is not driven by species-specific selection alone.

There are several factors that may underlie the compositional differences in the NCC and NEAC intestinal microbiomes. First, for more than 10 months during the year, the two populations encounter different habitats, as the NEAC ecotype is distributed in the pelagic waters of the Barents Sea while NCC remains more stationary in coastal waters (49). Although several 16S rRNA-based studies have reported limited effects of geographic location on the composition and diversity of the fish intestinal microbiome (32, 50), the Barents Sea has significantly lower temperatures (51) than Norwegian coastal waters (52). Temperature has been shown to have a significant impact on the intestinal microbiome in several studies, e.g., Senegalese sole (Solea senegalesis), Tasmanian Atlantic Salmon (Salmo salar), and mummichog (Fundulus heteroclitus) (5, 53, 54), but not in all cases (e.g., Atlantic salmon) (55). Second, the ecotypes were sampled during different seasons: NCC Lofoten during summer (August) and NEAC during winter/early spring (March). Nonetheless, a lack of difference between NCC Lofoten (August) and NCC Oslo Fjord (May) suggests that seasonality is unlikely to fully explain the observed differences between NEAC and NCC. Third, the ecotypes show different feeding behaviors; while the NEAC may perform vertical movements down to 500 m during foraging and spawning migrations from the Barents Sea (42, 56, 57), NCC mainly occupy shallow and warmer coastal and fjord waters (58). These behaviors expose the two ecotypes to different sources of food, with NEAC predominantly eating capelin and herring (48) and NCC living on a more diverse diet, including crustaceans, fish, and even seaweeds (34, 39). Diet has been shown to influence the composition of the intestinal microbiome in several fish species (9, 10, 13, 53, 59, 60). Finally, the Barents Sea has a high microbial biodiversity compared to coastal areas (61). The specific bacterial load in the surrounding waters also influences the intestinal microbiome composition in fish, including Atlantic cod (3, 4). Nonetheless, because these different environmental and behavioral factors are correlated, it is unclear which of these parameters contributes the most to the observed differences in the intestinal microbiome composition between these ecotypes.

Comparing two spatially separated coastal Atlantic cod populations, metagenomic shotgun data revealed no strain-level differentiation (30). In this study, we found specific SNV variants among the most abundant bacterial species that were associated with single species or specific Atlantic cod ecotypes. This indicates that NEAC harbor different strains of *P. iliopiscarium* than those identified in the NCC ecotype and the other gadoid species. Our current study encompasses a significantly greater geographical area and broader range of taxonomical samples than the earlier coastal comparison (30–32), and is indicative of strain-level variation at larger comparative scales. In line with Riiser et al. (30), this study shows that such strain-level differences cannot be detected using 16S rRNA techniques alone, and that metagenomic shotgun sequencing is currently the most accurate approach to detect strain-level spatial variation in the marine environment.

Most striking among the comparisons of gadoid species were the microbiome differences observed in NEAC, northern silvery pout, and poor cod compared to NCC and Norway pout. Several bacterial species that drive this differentiation are of particular interest. First, two bacterial species, *Mucispirillum* sp. and *Brevinema* sp., are almost exclusively detected in the intestinal microbiomes of NEAC and northern silvery pout. Nonetheless, these genera are represented by a single species in the *RefSeq* database (62) (accessed 10 January 2019) and hence little is known. Brevinema andersonii (order *Brevinematales*) was originally identified in short-tailed shrews (Blarina brevicauda) and white-footed mice (Peromyscus leucopus) and was found to be unable to grow below 25°C (63). *Brevinema* spp. have previously been identified in Atlantic cod (32) and in Atlantic salmon (64). Mucispirillum schaedleri (order *Deferribacterales*) is a mucosa-associated member of the intestinal microbiome in terrestrial animals such as pigs, goats, and rodents, where it is thought to be involved in mucus production through expression of lectins, important components in the innate immune response (65, 66). Nevertheless, the distant relationship between Atlantic cod and these terrestrial hosts and the availability of only single reference genomes for *Mucispirillum* and *Brevinema* strongly suggest that the representatives found here are related but novel species with different intestinal ecologies and physiologies. Second, both NEAC and northern silvery pout contain significant fractions of *Brachyspira* spp., previously identified as dominant members in the gut of the carnivorous marine fish species mahi mahi (Coryphaena hippurus) (12, 67). *Brachyspira* spp. are known as intestinal pathogens in pigs and humans (68, 69), although recent studies show that *Brachyspira* spp. are more widespread in the wildlife community than previously thought, including in freshwater (70). The ecology of *Brachyspira* in the marine environment is unclear, although an association with the carnivorous diet of mahi mahi and NEAC may suggest that the diet of northern silvery pout also has a considerable carnivorous component. Third, poor cod is the only species with considerable abundance of Enterovibrio norvegicus (Table S11). This bacterium within the *Vibrionaceae* family was isolated from the intestines of cultured turbot (Scophthalmus maximus) larvae in Norway and classified as a novel species phenotypically similar to the *Vibrio* genus (71). Interestingly, poor cod also host the highest abundance of *Vibrio* spp. among the fish species in this study (Table S11). Other *Enterovibrio* species have been found in association with diseased corals (72) and internal organs of cultured fish species in the Mediterranean Sea (73–75). However, little is known about the function of this relatively novel genus in fish intestines.

Given the observations of species-specific selection for a similar microbiome in various teleosts and range of habitats (13, 14, 16–19, 29, 33), the diverse microbiomes within and among gadoid species may suggest that their intestinal communities could be more easily modulated by external factors. At this stage, limited sampling across various fish taxa and the lack of comparative approaches leave reasons for such diverse communities speculative. Nonetheless, it is interesting to note that all gadoids have an unusual adaptive immune system in which there is loss of MHC II, CD4, and invariant chain (Ii) and a range of innate (TLR) and MHC I immune-gene expansions (23, 24). There are significant correlations between immune genes and the vertebrate microbiome (76, 77), and it has been hypothesized that adaptive immunity has evolved to help maintain a complex community of beneficial commensal bacteria (78). Indeed, studies of wild-type zebrafish and knockout zebrafish without a functional adaptive immune system suggest that adaptive immunity increases the strength of host filtering of potential fish-associated microbes (22). The unusual adaptive immune system of gadoids may therefore affect the strength of coevolutionary associations within their microbiomes.

In conclusion, based on metagenomic shotgun sequencing, we here characterize the intra- and interspecific community compositions among two ecotypes of Atlantic cod and three related fish species in the Gadidae family. Several of these fish species harbor unique, and possibly novel, bacterial species. We identify a complex pattern of diversity with significant differences between the Atlantic cod ecotypes and variable interspecific patterns of variation. Although most species and ecotypes yield different communities, those found in coastal cod (NCC) and Norway pout are not significantly diverged, indicating that ecological niche plays an important role in determining the intestinal microbiomes in these gadoid species.

## MATERIALS AND METHODS

### Sample collection.

Northeast Atlantic cod (*Gadus morhua*) (NEAC, 10 individuals) were collected in Lofoten (N68.0619167, E13.5921667) in March 2014 and Norwegian coastal cod (*Gadus morhua*) (NCC, 10 individuals) at the same location in August 2014 (Fig. 1a; see Table S1 in the supplemental material). NCC (2 individuals) were also collected in the Oslo Fjord (N58.9125100, E9.9202624 and N59.8150006, E10.5544914). Norway pout (*Trisopterus esmarkii*, 4 individuals), poor cod (*Trisopterus minutus*, 5 individuals), and northern silvery pout (*Gadiculus thori*, 3 individuals) were collected in the inner Oslo Fjord in May 2015 (Table S1). All fish specimens were collected from wild populations. A 3-cm-long part of the hindgut (immediately above the short, wider rectal chamber) was aseptically removed postmortem by scalpel and stored in 70% ethanol. The samples were frozen (–20°C) for long-term storage. Relevant metadata such as length, weight, sex, and maturity were registered. As we strive to reduce the impact of our sampling needs on populations and individuals, samples were therefore obtained as a by-product of conventional business practices. Specimens were caught by commercial vessels, euthanized by local fishermen, and intended for human consumption. Samples were taken postmortem and no scientific experiments have been performed on live animals. This sampling follows the guidelines set by the “Norwegian consensus platform for replacement, reduction and refinement of animal experiments” (79) and does not fall under any specific legislation in Norway, requiring no formal ethics approval.

### Sample preparation and DNA extraction.

Intestinal samples were split open lengthwise before the combined gut content and mucosa were gently removed using a sterile disposable spatula. Each individual sample was washed in 500 μl 100% ethanol (EtOH) and centrifuged before the ethanol was allowed to evaporate, after which dry weight was measured before proceeding to DNA extraction. DNA was extracted from between <10 and 300 mg dry weight of gut content using the MoBio Powersoil HTP 96 Soil DNA isolation kit (Qiagen, Valencia, CA, USA) according to the DNA extraction protocol (v. 4.13) utilized by the Earth Microbiome Project (80). DNA was eluted in 100 μl elution buffer and stored at −20°C. Due to high methodological consistency between biological replicates in previous experiments, only one sample was collected per fish (32).

### Sequence data generation and filtering.

Quality and quantity of the DNA was measured using a Qubit fluorometer (Life Technologies, Carlsbad, CA, USA) and normalized by dilution. DNA libraries were prepared using the Kapa HyperPlus kit (Roche Sequencing, Pleasanton, CA, USA) and paired-end sequenced (2 × 125 base pairs) on an Illumina HiSeq2500 using the HiSeq SBS V4 chemistry with dual indexing in two independent sequencing runs. Read qualities were assessed using FastQC (81) before adapter removal, singleton read identification, deduplication, and further read quality trimming was performed using Trimmomatic (ver. 0.36) (82) and PRINSEQ-lite (ver. 0.20.4) (83) (Table S2). PhiX, host, and human sequences were removed by mapping reads to the phiX reference genome (GenBank: J02482.1), the Atlantic cod genome assembly (gadMor 2) (this applied to all the fish species) (84), and a masked version of the human genome (HG19) (85) using BWA (ver. 0.7.13) (86) or BBMap (ver. 37.53) (87) (JGI) with default parameters and discarding matching sequences using *seqtk* (ver. 2012.11) (88). All sequence data have been deposited in the European Nucleotide Archive (ENA) under study accession number PRJEB31095.

### Taxonomic profiling.

Taxonomic classification of quality trimmed and filtered metagenomic paired-end reads was performed using Kaiju (ver. 1.5.0) (89) (“greedy” heuristic approach, -e 5), with the NCBI *nr* database (release 84) (including proteins from fungal and microbial eukaryotes) as reference (62). Counts of sequences successfully assigned to orders and species were imported into RStudio (ver. 1.1.383) (90), based on R (ver. 3.4.2) (91), for further processing. Filtering of the most abundant bacterial orders for visualization was based on a minimum relative abundance threshold of 1% of the total number of sequences per library (the threshold ranged from 5,933 to 95,146 depending on the sample size). Similarly, filtering of the most abundant bacterial species was based on a minimum relative abundance threshold of 2% of the total number of sequences per library (the threshold ranged from 6,548 to 190,294 depending on the sample size). Any taxon not exceeding this threshold in at least one (order-level) or two (species-level) samples was removed. All filtering was based on the R package genefilter (ver. 1.62.0) (92). Final results were visualized using the R package ggplot (ver. 2.2.1) (93). Note that, based on a recent reclassification (94), we refer to the reference strain Photobacterium phosphoreum ANT-2200 (accession number GCF_000613045.2) as Photobacterium kishitanii (Table S3).

### Sequence variation analysis.

In order to assess the heterogeneity of the most abundant bacteria in the fish species, we analyzed the sequence variation in the two genomes with the highest mean relative abundance over all fish species and ecotypes, Photobacterium kishitanii and Photobacterium iliopiscarium. Paired-end reads from each individual fish were mapped to the reference genomes (Table S3) using the Snakemake workflow (95) of anvi’o (ver. 5.1) (96) with default parameters in the “all-against-all” modus (with anvi-profile –min-coverage-for-variability 20). Samples of low coverage, restricting detection of SNVs in anvi’o, were excluded from the variation analysis. For each individual sample, variable sites were identified, and the mean number of these per 1,000 bp was calculated (variation density). A variable site required a minimum coverage of 20×. Next, variable sites with a minimum of 20× coverage in all samples were defined as single nucleotide variants (SNVs, anvi-gen-variability-profile –min-occurrence 1 –min-coverage-in-each-sample 20). Coverage, variation density, and SNV profiles were plotted in RStudio following the R script provided by anvi’o (97). The anvi’o SNV output was converted to .vcf format using a custom-developed script (https://github.com/srinidhi202/AnvioSNV_to_vcf), and the resulting .vcf files were used in a principal-component analysis (PCA) to test for population differences as implemented in smartpca (ver. 6.1.4) (EIGENSOFT) (98).

### Statistical analysis.

Although included in data visualization, the Oslo Fjord NCC samples were excluded from statistical analysis due to low sample size (*n* = 2). Within-sample diversity (alpha diversity) was calculated using the diversity function in the R package vegan (ver. 2.4-1) (99) based on Shannon, Simpson, and inverse Simpson indices calculated from nonnormalized order-level read counts (Table S4). Differences in alpha diversity were studied using linear regression. The “optimal model” (the model that best describes the individual diversity) was identified through a “top-down” strategy, including all covariates (Table S5) except weight, which highly correlated with length (*r* = 0.95), and selected through *t* tests. Model assumptions were verified through plotting of residuals. Differences in bacterial community structure (beta diversity) between the fish species or ecotypes were visualized using nonmetric multidimensional scaling (NMDS) plots based on the Bray-Curtis dissimilarity calculated from order-level sequence counts. Next, pairwise differences in beta diversity between the fish species or ecotypes were tested using permutational multivariate analysis of variance (PERMANOVA) in the R package pairwise.adonis (ver. 0.1) (100), a wrapper for the adonis functions in vegan (ver. 2.4-1), based on Bray-Curtis dissimilarity calculated from order-, genus- and species-level sequence counts. The package pairwise.adonis was run with 20,000 permutations, and *P* values were adjusted for multiple testing using the Holm method (101). Adjusted *P* values of <0.05 indicate statistical significance. PERMANOVA assumes the multivariate dispersion in the compared groups to be homogeneous; this was verified (*P* > 0.05) using the betadisper function (vegan) (Table S6). Similarity percentage (SIMPER) procedure implemented in vegan was used to quantify the contribution of individual orders to the overall Bray-Curtis dissimilarity between the species/ecotypes. All beta diversity analyses were based on sequence counts normalized using a common scaling procedure, following McMurdie and Holmes (102). This involves multiplying the sequence count of every unit (e.g., order) in a given library with a factor corresponding to the ratio of the smallest library size in the data set to the library size of the sample in question. Normalization using this procedure effectively results in library scaling by averaging an infinite number of repeated subsamplings. We used chi-squared statistics, as implemented in smartpca (98), to test for significant differences in the distributions of SNVs per reference genome while correcting for multiple testing using sequential Bonferroni (101).

### Data availability.

The data set generated and analyzed for this study is available in the European Nucleotide Archive (ENA) under study accession number PRJEB31095.

## Supplementary Material

Supplemental file 1
